# Weight Loss and Mortality: What Does the Evidence Show?

**DOI:** 10.1371/journal.pmed.0020181

**Published:** 2005-06-28

**Authors:** Meir Stampfer

## Abstract

A study in this month's PLoS Medicine concludes that, "deliberate weight loss in overweight subjects, without known co-morbidities, may be hazardous in the long term." Stampfer questions whether the data really support such a conclusion.

Randomized trials and observational studies have conclusively shown a marked improvement in several cardiovascular risk factors when obese individuals lose weight, including decreases in blood pressure, and improvements in the lipid profile and glucose metabolism. These results are closely consistent with observational data linking weight gain to worsening of these cardiovascular risk factors, and to their natural sequelae, clinical cardiovascular events. Obesity is also strongly associated with increased risk of cancer at various sites. With such compelling evidence of the adverse effects of obesity and overweight [1–4], why has there been a continuing controversy about its association with overall mortality?

In their paper in this month's *PLoS Medicine*, Jaakio Kaprio and colleagues conclude that “deliberate weight loss in overweight subjects without known co-morbidities may be hazardous in the long term” [5]. Do the data really support such a conclusion? An examination of this paper illustrates several of the complexities in approaching this seemingly simple question.

## Evidence from Epidemiological Studies

Epidemiologic studies of the relation between overweight and mortality typically must address three principal concerns [6]. First, in many populations, cigarette smokers tend to be leaner than nonsmokers. Because cigarette smoking is such a strong risk factor for mortality, failure to adjust for this adequately can lead to confounding, with the erroneous conclusion that leanness carries increased risk of death. Statistical adjustment for smoking is often insufficient to account for this difficulty. Smoking can lead to medical conditions, sometimes sub-clinical, that are associated with decreased body weight, such as chronic obstructive pulmonary disease. The presence of symptoms or diagnosed conditions may induce smokers to quit. Moreover, the intensity of smoking is related to both risk of death and body mass index. For these reasons, the best way to assess the impact of overweight on risk of mortality is simply to exclude current and past smokers. Kaprio and colleagues' study differentiated only current smoker or nonsmoker. Thus, never-smokers were included in the same category as past smokers, regardless of how much the past smokers had smoked or their reasons for quitting.

A second difficulty in some epidemiologic studies is the inclusion of intermediary factors as co-variates. Weight loss improves hypertension and diabetes, so including these as co-variates would tend to attenuate the apparent benefit of weight loss. In the present study, the authors appropriately excluded people with diabetes, and adjustment for hypertension appeared to have little impact.

The third and most difficult issue in studies of overweight and mortality is reverse causation, the impact of disease on body weight. This can occur either through the biological impact of a condition (diagnosed or preclinical) or as an inducement to attempt to lose weight as a means to improve health. The authors' keen recognition of this problem provides a significant strength to the present study. The authors appropriately excluded individuals with a wide range of conditions to identify an apparently healthy cohort. This critical step is often ignored. Sometimes, investigators also exclude deaths that occur in the first few years after follow-up, to reduce the impact of reverse causation. Such lagged analyses can be helpful, but some chronic conditions of long duration, such as depression, chronic lung disease, and heart failure (conditions that often may not reach the level of clinical diagnosis) can lead to lower body weight and higher mortality risk. Hence, that strategy (not employed by Kaprio and colleagues) may not fully avoid the problem.

## The Difficulties of Assessing the Effects of Intention to Lose Weight

As a further step toward reducing the potential bias of the impact of disease on body weight, the authors identified individuals with intent to lose weight. Unintended weight loss is well known as an ominous clinical sign, usually signifying a serious illness. Just over a third of the overweight subjects in the Kaprio cohort had expressed intent to lose weight at baseline in 1975. It is interesting to note that despite this intention, as a group, the median weight change in the ensuing six years was a gain of 0.33 kg/m^2^, almost identical to the weight change in the group that had not expressed intent to lose weight (gain of 0.31 kg/m^2^). Thus, this study cannot fairly be characterized as an assessment of intentional weight loss and its subsequent effect on mortality. Because the changes in weight are so similar in these two groups, it is implausible to attribute the weight loss in the intent-to-lose group to that intention. These findings render the results particularly difficult to interpret.

Other important limitations include the very small number of endpoints—only 268 total deaths in the cohort. When further subdivided according to intention to lose weight, and categories of weight change, the numbers are far from sufficient for reliable estimates. For example, the main conclusions are based on the subgroup of those with intent to lose weight who actually lost weight; this group had only 42 deaths, of which ten were violent. Another related difficulty is that this cohort, though overweight, is not drastically obese. The median body mass index (BMI) was 26.7 kg/m^2^, and fewer than 10% of people had a BMI greater than 30. With such a narrow range of BMI, coupled with the very small changes in weight during the six years of initial follow-up, even a very large study would not have sufficient power to detect plausible relative risks associated with weight change.

## What Advice Should Be Given?

Given the well-known adverse metabolic consequences of overweight and obesity, the strong links with cardiovascular disease and several other serious illnesses, and the absence of any plausible adverse biologic consequences of maintaining normal weight (BMI 19 to 25), it seems prudent to continue advising adults to seek to maintain a weight within that range. The results from Kaprio et al, although they raise questions about the effects of weight loss, do not cast serious doubt on that advice.

## Abbreviation

BMI: body mass index
